# Supplementary figures and images for: Combinatorial interactions between viral proteins expand the potential functional landscape of the tomato yellow leaf curl virus proteome
Source: PLoS Pathog. 2022 Oct 18;18(10):e1010909. doi: 10.1371/journal.ppat.1010909 (PMC9633003; doi:10.1371/journal.ppat.1010909)

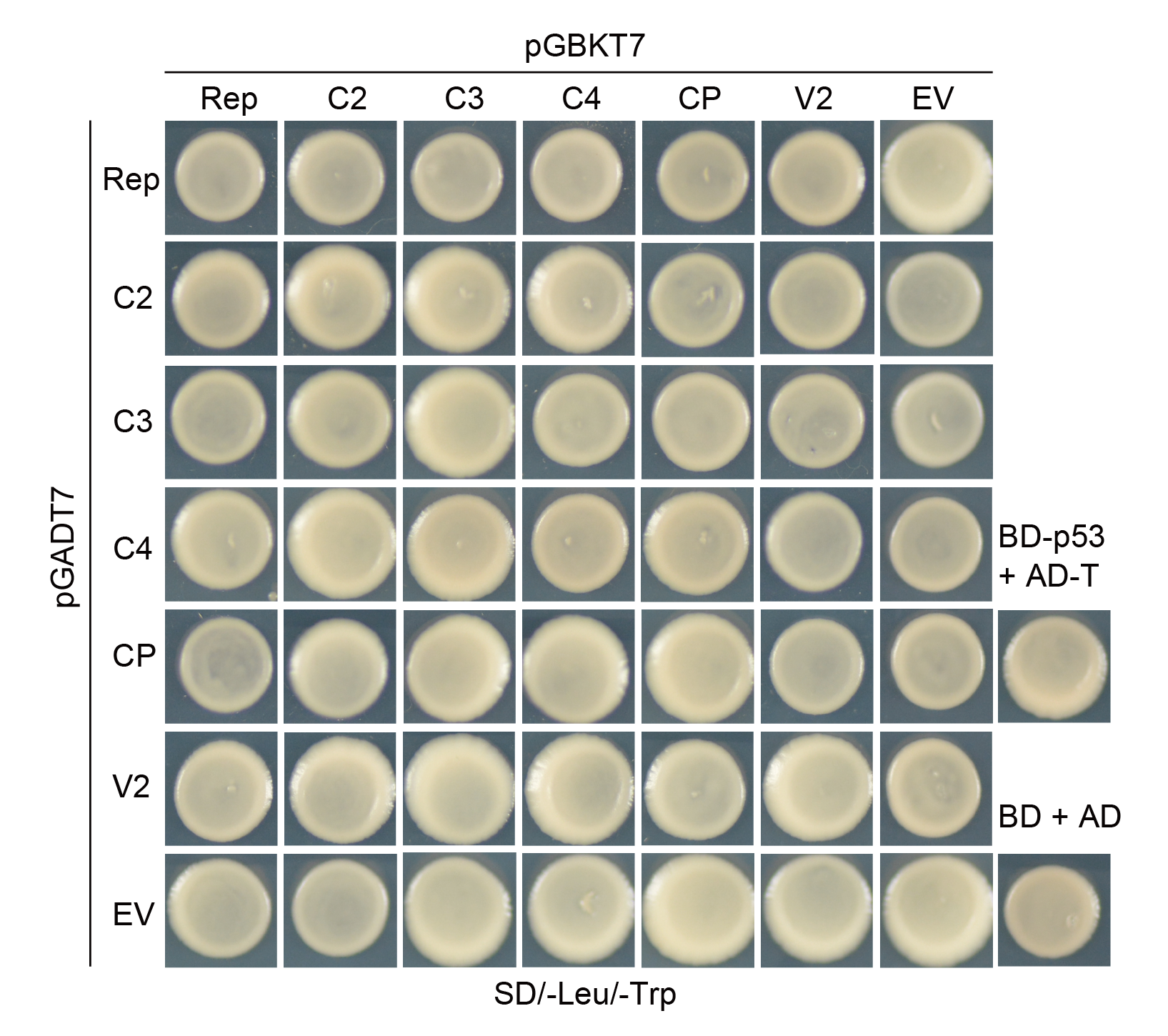

Supplement: S1 Fig — The minimal synthetic defined (SD) medium without leucine (Leu) and tryptophan (Trp) was used to select co-transformants. (TIF) [file ppat.1010909.s001.tif]

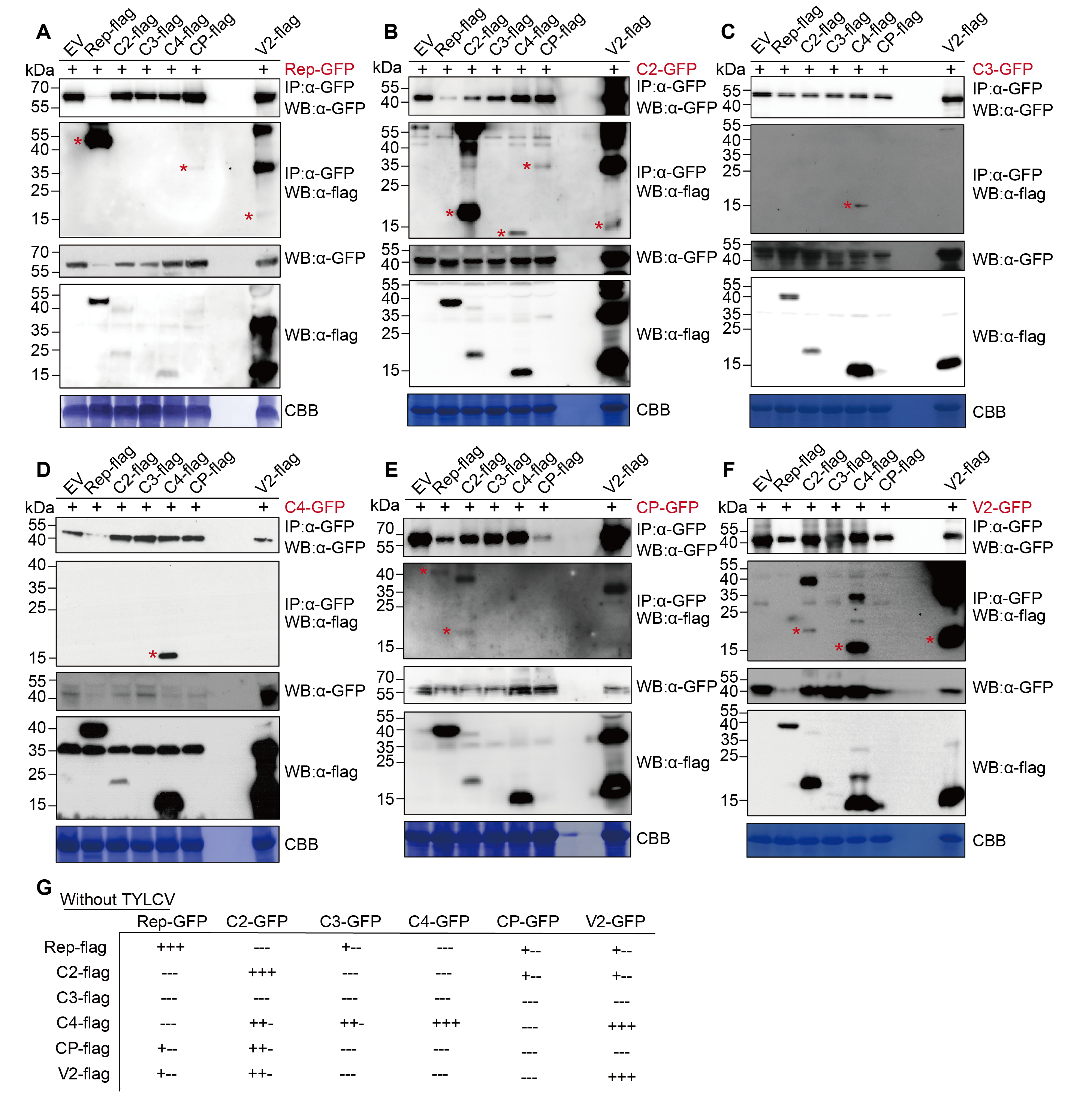

Supplement: S2 Fig — (A-F) Co-immunoprecipitation (co-IP) assays of Rep-, C2-, C3-, C4-, CP- and V2-FLAG with Rep- (A), C2- (B), C3- (C), C4- (D), CP- (E) or V2-GFP (F) following transient expression in N. benthamiana leaves. IB: immunoblotting, IP: immunoprecipitation, CBB: Coomassie brilliant blue. Molecular weight of Rep-, C2-, C3-, C4-, CP-, and V2-GFP is 65, 42, 43, 38, 57, and 40 kDa, respectively; molecular weight of Rep-, C2-, C3-, C4-, CP-, and V2-FLAG are 41, 15, 16, 11, 30, and 14 kDa, respectively. Asterisks indicate the expected band for each protein. (G) Summary table containing the results of all co-IP replicates performed in the absence of the virus. Column headings indicate the viral protein used as bait; row headings indicate prey proteins. (TIF) [file ppat.1010909.s002.tif]

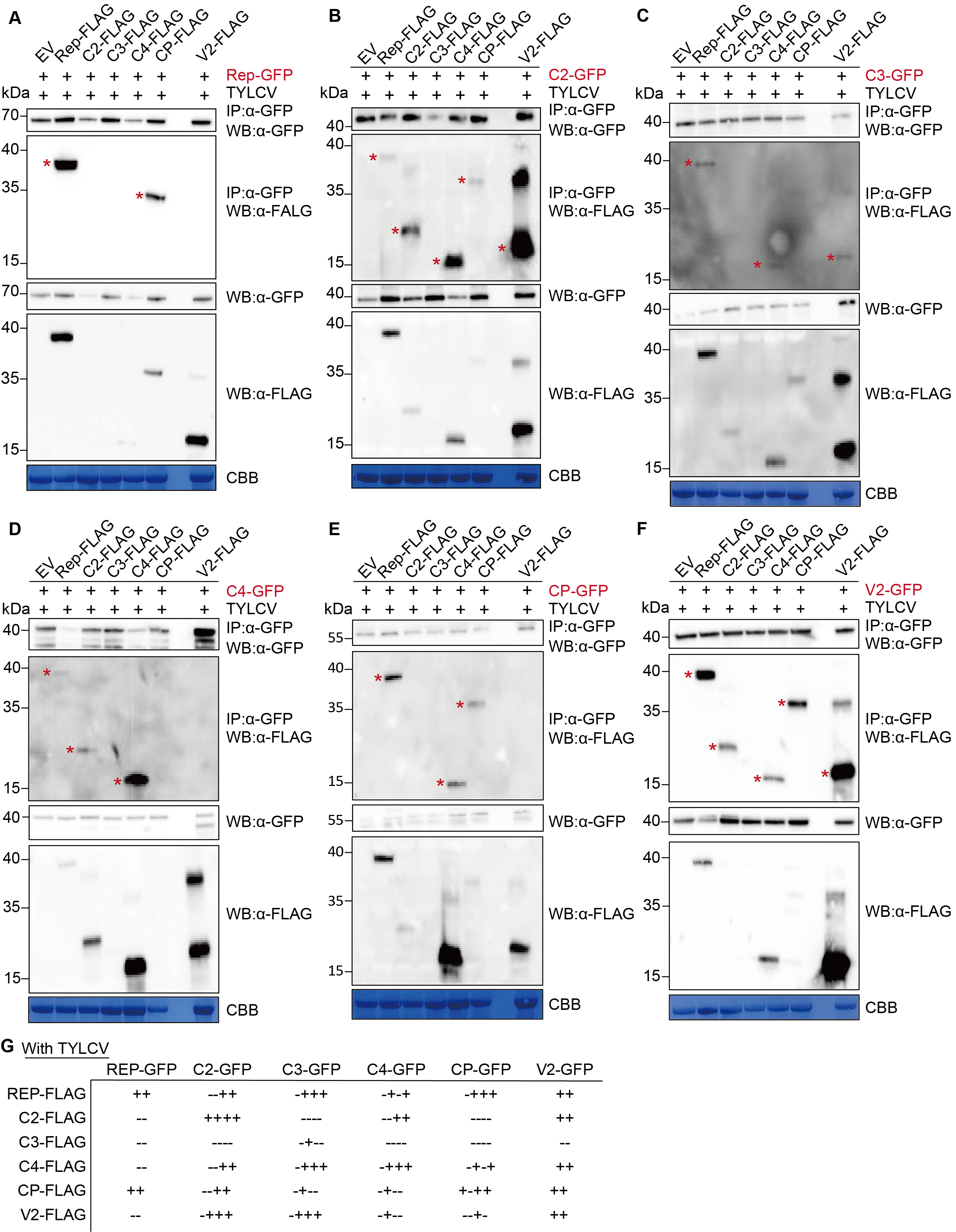

Supplement: S3 Fig — (A-F) Representative co-immunoprecipitation (co-IP) assays of Rep-, C2-, C3-, C4-, CP- and V2-FLAG with Rep- (A), C2- (B), C3- (C), C4- (D), CP- (E) or V2-GFP (F) following transient expression in N. benthamiana leaves in the presence of the virus. IB: immunoblotting, IP: immunoprecipitation, CBB: Coomassie brilliant blue. Molecular weight of Rep-, C2-, C3-, C4-, CP- and V2-GFP is 65, 42, 43, 38, 57, and 40 kDa, respectively; molecular weight of Rep-, C2-, C3-, C4-, CP- and V2-FLAG are 41, 15, 16, 11, 30, and 14 kDa, respectively. Asterisks indicate the expected band for each protein. (G) Summary table containing the results of all co-IP replicates performed in the presence of the virus. Column headings indicate the viral protein used as bait; row headings indicate prey proteins. (TIF) [file ppat.1010909.s003.tif]

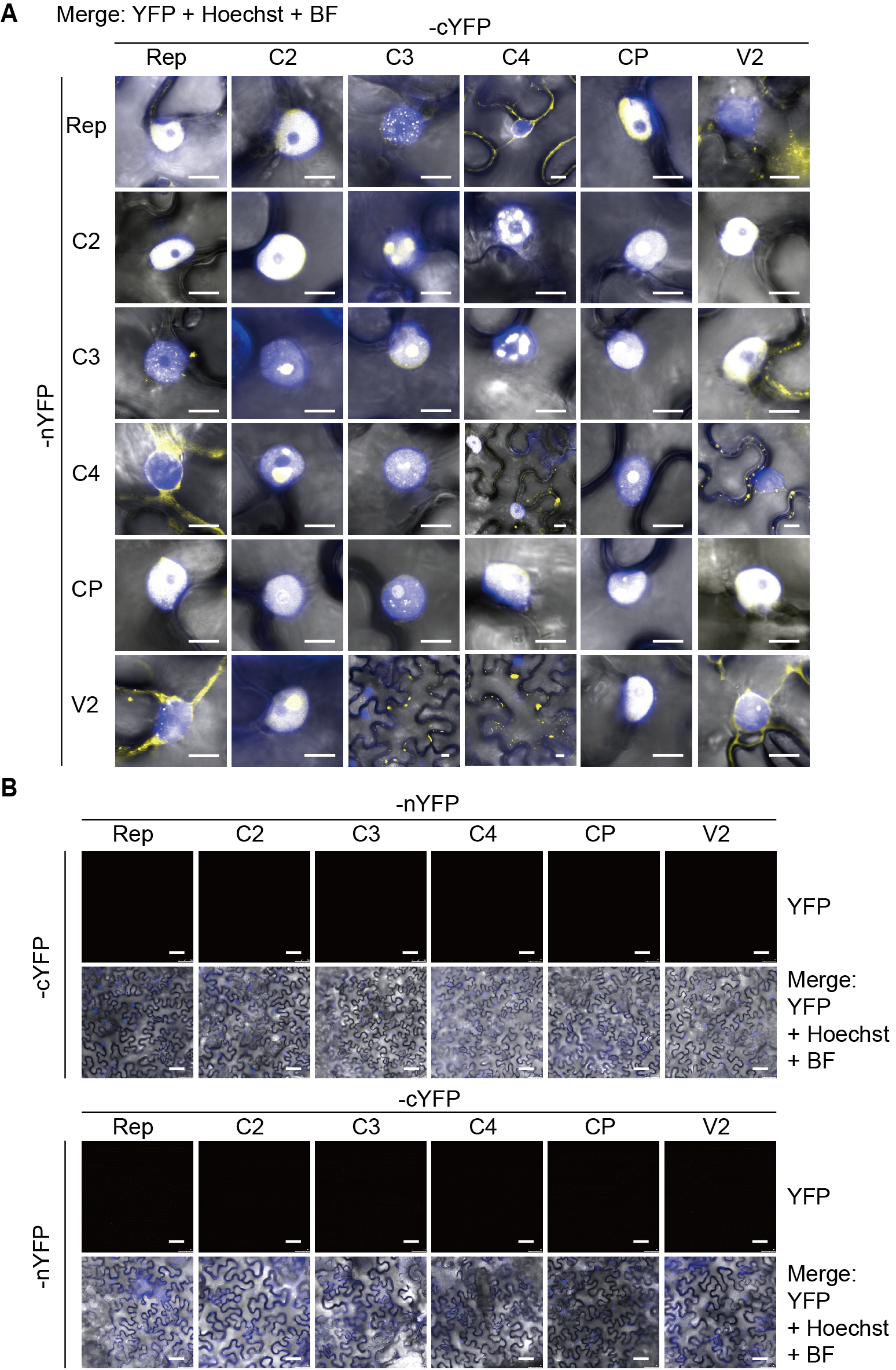

Supplement: S4 Fig — nYFP: N-terminal half of the YFP; cYFP: C-terminal half of the YFP; BF: bright field. Images were taken at 2 days post-infiltration (dpi). Scale bar = 10 μm in (A) or 50 μm in (B). This experiment was repeated at least four times with similar results. (TIF) [file ppat.1010909.s004.tif]

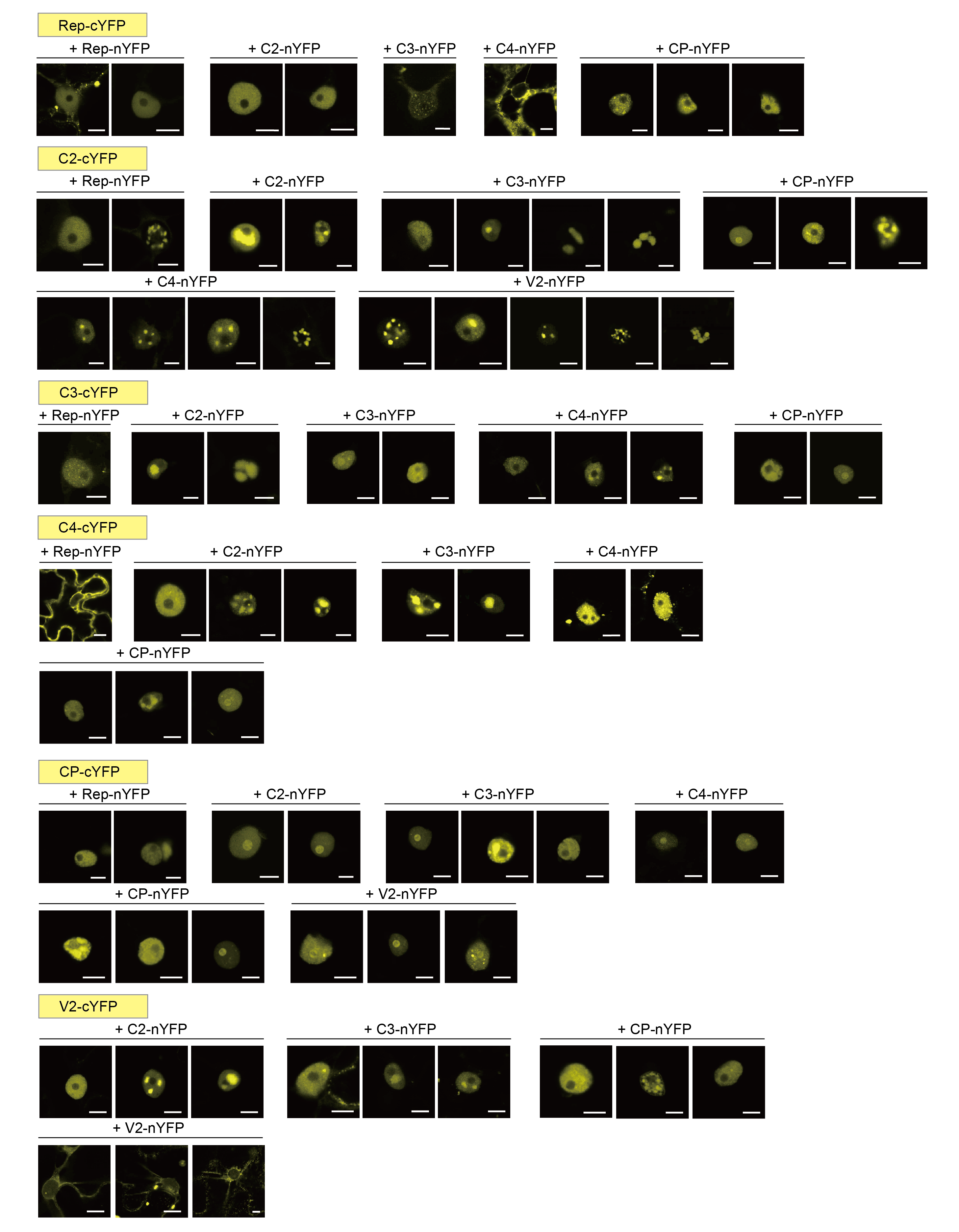

Supplement: S5 Fig — nYFP: N-terminal half of the YFP; cYFP: C-terminal half of the YFP. Images were taken at 2 days post-infiltration (dpi). Scale bar = 10 μm. This experiment was repeated at least four times with similar results. (TIF) [file ppat.1010909.s005.tif]

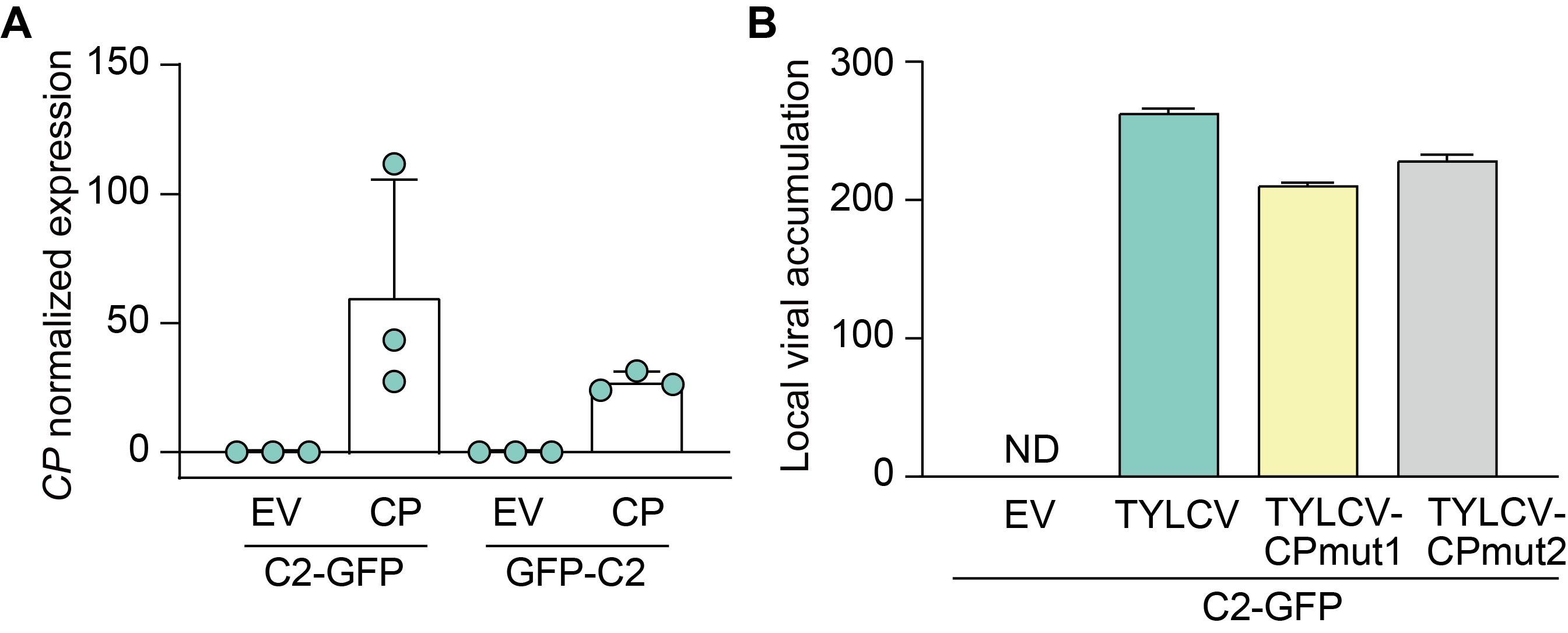

Supplement: S6 Fig — (A) Accumulation of the CP transcript (from Fig 3C), measured by RT-qPCR. NbEF1α was used as the normalizer. Values represent the mean of three plants. Error bars represent SD. (B) Accumulation of viral DNA in samples from Fig 3D. ITS was used as the normalizer. Values represent the mean of three plants. Error bars represent SD. (TIF) [file ppat.1010909.s006.tif]

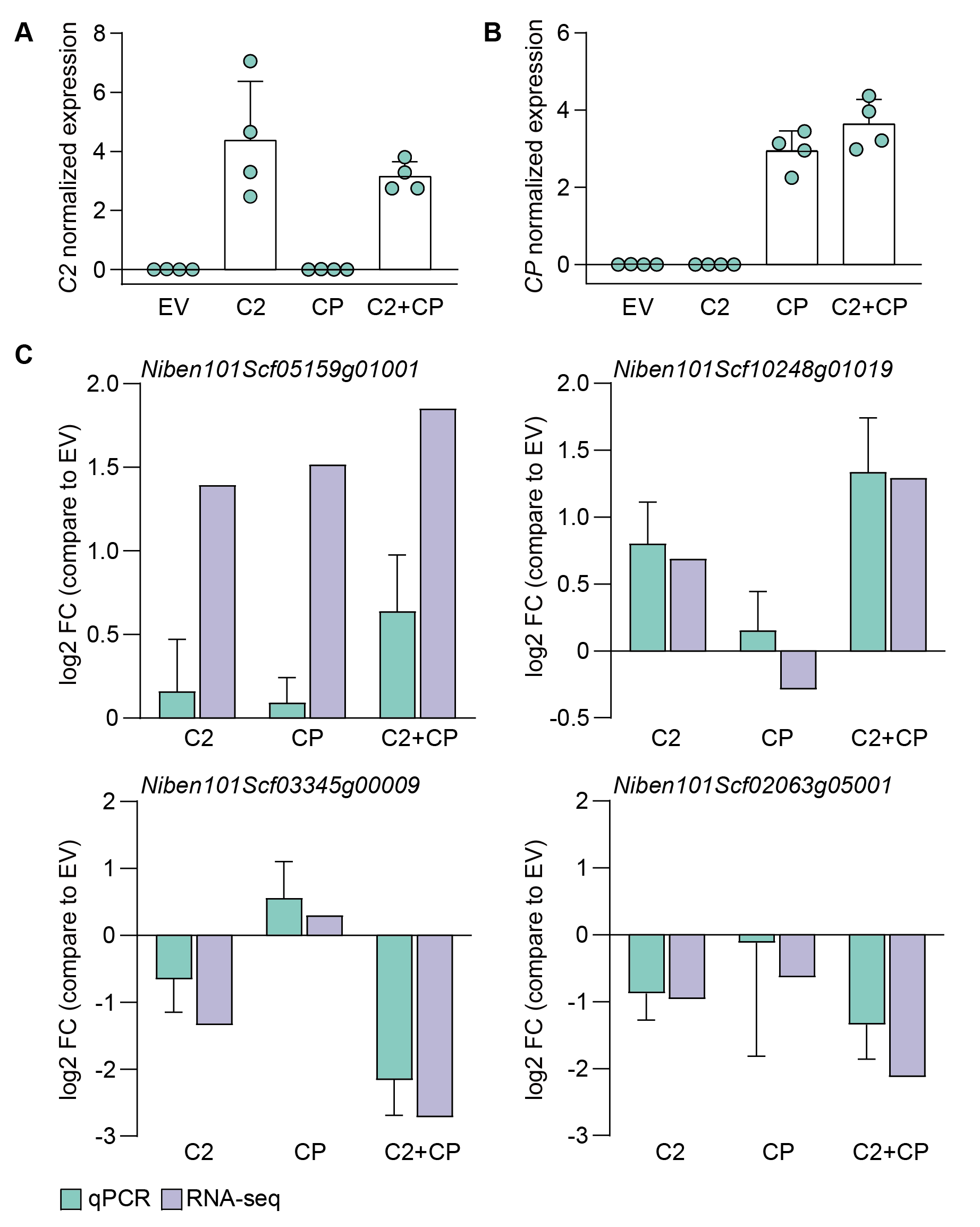

Supplement: S7 Fig — (A-B) C2 and CP transcript accumulation, measured by RT-qPCR. Expression values are relative to NbACT2. Results are the mean of four biological replicates. Error bars represent SD. (C) Comparison of the accumulation of transcripts of selected DEGs in the RNA-seq data and as measured by RT-qPCR. Expression values are the mean of log2 FC, relative to EV, from four biological replicates. NbACT2 was used as the normalizer. FC: fold change; EV: empty vector. (D) Expression of selected DEGs upon expression of C2, C2-GFP, GFP-C2 in the presence and absence of CP in N. benthamiana leaves. Samples expressing CP or empty vector (EV) are used as control. Expression values are the mean of at least three biological replicates. Error bars represent SD. Asterisks indicate a statistically significant difference (*: p<0.05) according to a two-tailed comparison t-test. NbACT2 was used as the normalizer. (TIF) [file ppat.1010909.s007.tif]

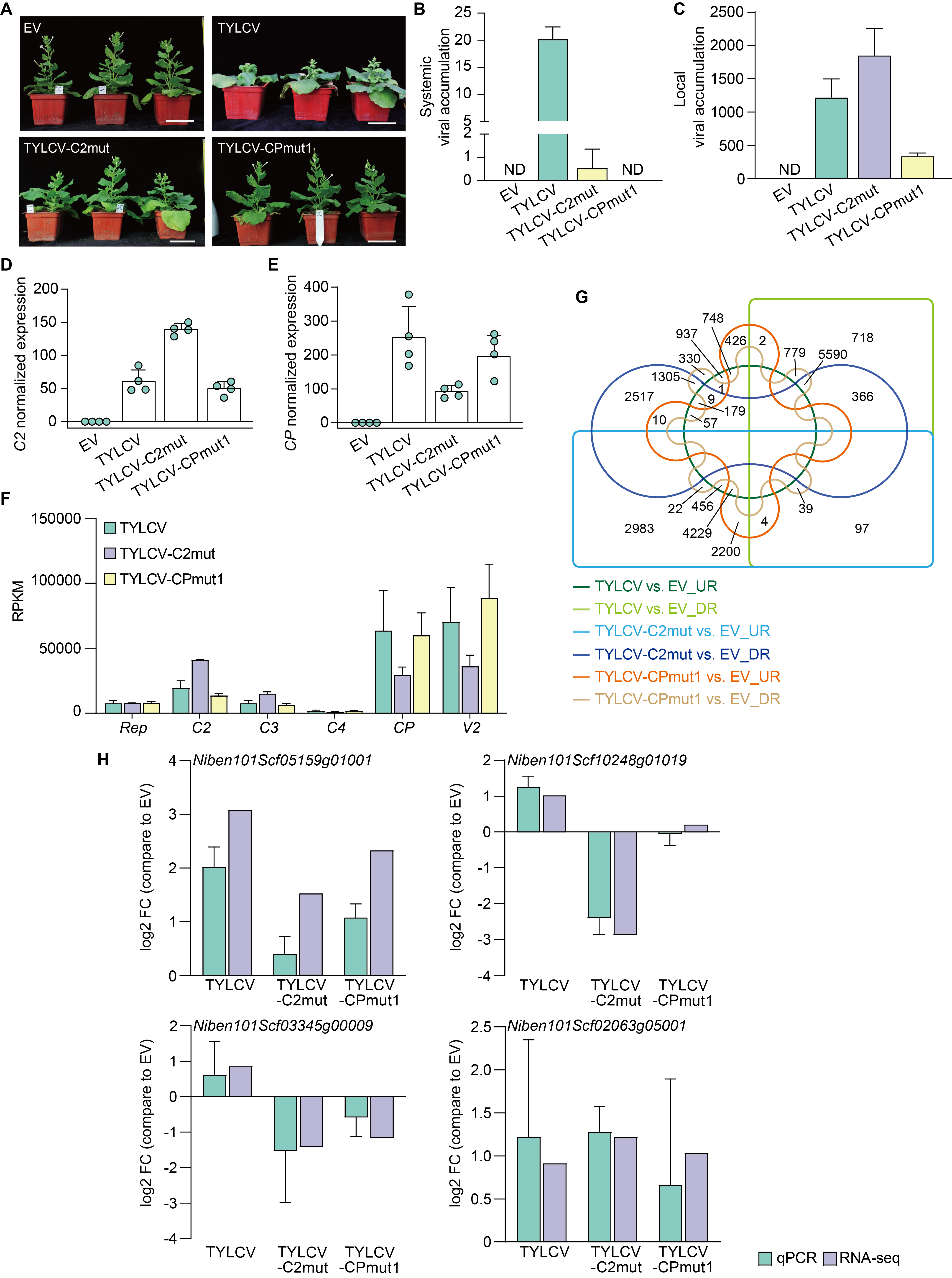

Supplement: S8 Fig — (A) Symptoms in N. benthamiana plants inoculated with TYLCV WT or C2-/CP-null mutants (TYLCV-C2mut and TYLCV-CPmut1, respectively), or inoculated with empty vector (EV) as negative control. Pictures were taken at 21 days post-inoculation (dpi). Scale bar: 10 cm. (B) Viral DNA accumulation in systemic infections in N. benthamiana plants measured by qPCR. Values are the mean of three independent biological replicates. Error bars represent SD. Samples were taken at 21 dpi. ITS was used as the normalizer. (C) Viral DNA accumulation in N. benthamiana leaves infiltrated with TYLCV WT or C2-/CP-null mutants (TYLCV-C2mut and TYLCV-CPmut1, respectively), or transformed with empty vector (EV) as negative control. Samples were taken at 2.5 days post-inoculation (dpi). ITS was used as the normalizer. Values represent the mean of six plants. Error bars represent SD. (D-E) C2 and CP transcript accumulation. Expression values are relative to NbACT2. Results are the mean of four biological replicates. Error bars represent SD. (F) Expression of TYLCV genes in the different samples as detected by RNA-seq. RPKM: reads per kilobase of transcript per million mapped reads. (G) Venn diagram of the subsets of up- and down-regulated genes in the samples infected with TYLCV WT or C2-/CP-null mutants (TYLCV-C2mut and TYLCV-CPmut1) compared to the empty vector control (EV). UR: up-regulated; DR: down-regulated. (H) Expression of selected DEGs. Expression values are the mean of log2 FC, relative to samples inoculated with the EV, from four biological replicates. NbACT2 was used as the normalizer. FC: fold change; EV: empty vector. (TIF) [file ppat.1010909.s008.tif]

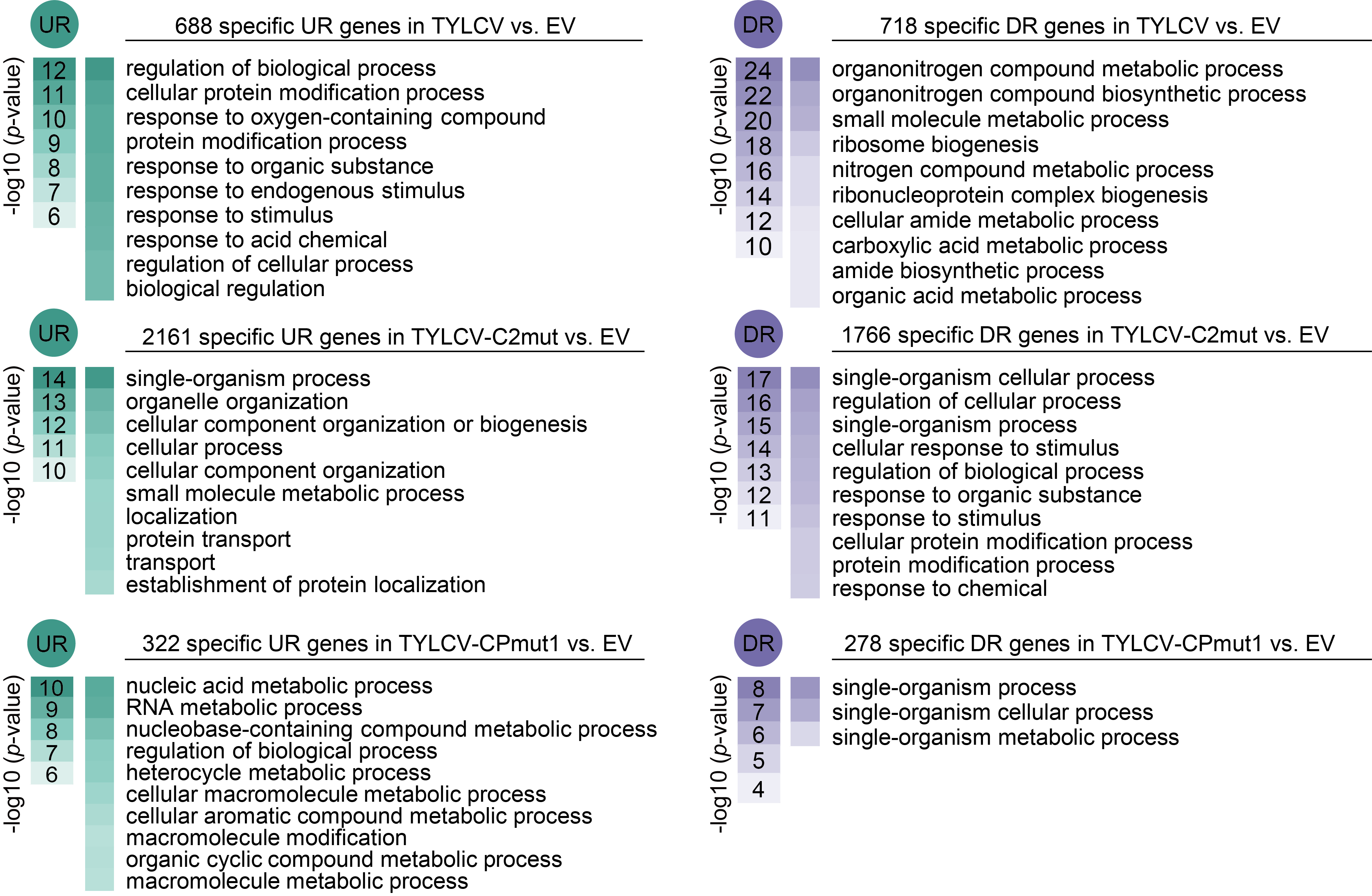

Supplement: S9 Fig — Gene Ontology (GO) categories from the Biological Process ontology enriched with a p-value<0.01 (up to top 10) are shown; functional enrichment analysis was performed using the orthologues in A. thaliana. The colour scale indicates the -log10 (p-value), showing the significance of GO term enrichment. For a full list, see S5 Table. (TIF) [file ppat.1010909.s009.tif]
