# Supplementary material for: Combinatorial interactions between viral proteins expand the potential functional landscape of the tomato yellow leaf curl virus proteome
Source: PLoS Pathog. 2022 Oct 18;18(10):e1010909. doi: 10.1371/journal.ppat.1010909 (PMC9633003; doi:10.1371/journal.ppat.1010909)
Supplement: Attachment — Submitted filename: Response to reviewers - 09-08-22.docx [file ppat.1010909.s018.docx]

**Response to Reviewers**

First of all, we would like to thank Editor and Reviewers for their comments and constructive criticism on our manuscript. Following their advice and suggestions, we have made a number of changes to text and figures, and honestly believe the resulting manuscript is improved and has gained clarity. For detailed answers to the Reviewers’ comments, please see the point-by-point response below.

**Part II – Major Issues: Key Experiments Required for Acceptance**

*Reviewer #1:*

*The findings are still in an early stage. Authors clearly showed that viral proteins relocalize in the presence of their partners or full infection. These relocalization correlated well with the significantly changed transcriptomes. However, these are nice observations without mechanistic understanding.*

> Our results indicate that viral proteins physically interact with one another in an intricate network; in addition, we show that the interaction among viral proteins, physical or functional, can lead to changes in subcellular localization and effect on the cell, and therefore potentially underlie an expansion of the functional landscape of the viral proteome. We believe this is an important concept and, in our opinion, the main message of this manuscript, and goes beyond the mechanistic understanding of the effect of one particular pairwise viral protein-protein interaction. Nevertheless, the molecular underpinnings and detailed functional consequences of specific viral protein-protein interactions are undoubtedly of interest and will definitely be explored as part of future work.

*Reviewer #2:*

*1. It is reasonable to say that viral genomes have reduced coding capacity in the abstract. In fact, though the most viruses have limited genome, they are used to encode viral proteins at their maximum extent.*

> We agree with the reviewer in that viruses have evolved to maximize the coding ability of their genomes (e.g. by containing overlapping open reading frames); however, since their genomes are extremely small (compared to those of free-living organisms), according to our understanding their coding capacity can still be considered small. Nevertheless, following the reviewer’s advice and in order to avoid misleading the readers, we have replaced “reduced coding capacity” by “reduced genetic space” in the abstract.

*2. Fig 2C, is GFP-C2 or C2-GFP functional?*

> Whether GFP-C2 or C2-GFP are functional is difficult to assess, since there is no obvious functional assay that could be used in a complementation assay. Nevertheless, C2-GFP fusions have been used in functional studies with other geminiviral C2 proteins (e.g. van Wezel et al., 2001; van Wezel et al., 2002; van Wezel et al., 2003; Tu et al., 2017). In order to clarify this point, we have added a cautionary note to the text (L201-203 in the version with tracked changes).

*Reviewer #3:*

*Title: a reader expects a lot from words like “functional landscape of viral proteome”. Actually, the best part of this MS is in the analysis of the pair C2 and CP. I suggest to tune-down the emphasis present in the title. And include the term geminivirus or TYLCV.
Actually, the core of this MS is in the chapter: “The C2/CP module specifically reshapes the host transcriptome”, while the other chapters could be revisited (and the figures reduced) in view of preparing the reader to this one.*

> Following the reviewer’s advice, we have modified the title, which is now more specific (“Combinatorial interactions between viral proteins expand the potential functional landscape of the tomato yellow leaf curl virus proteome”). We have also specifically mentioned the C2-CP interaction in the abstract.

Although we agree with the reviewer in that many of the results in the manuscript are devoted to unravelling the potential effect of the C2-CP interaction pair, we believe that the finding that the viral proteins interact in an intricate network of physical associations is the most important contribution of this work, and the C2-CP module serves as an example of what such interactions can result in. Nevertheless, we have extensively re-written and re-arranged the manuscript, following the reviewers’ advice, and hope the general flow and readability are improved.

*This is a MS where so many different approaches have been used, with results not always in agreement with each other. This can, and should, be clearly stated as the readers should be able to understand the expriments, without the need of reading in detail several other previous papers.
Lines 125-127 and Fig. 2A: the case of V2 should be discussed, it is not IN the nucleus.*

> We have now extensively expanded the initial section to explain the approaches and results in detail, as well as to indicate and discuss seemingly discordant results (in particular, see sections “Viral proteins form complexes in the plant cell” and “The viral coat protein is required and sufficient to modify the subcellular localization of the virus-encoded C2 protein”).

V2 can be detected in the nucleus weakly in the nucleoplasm and strongly in the Cajal body (see Fig. 3 and Wang et al., 2020, *eLife*); however, a high accumulation of V2 appears associated to the endoplasmic reticulum (including a clear distribution around the nucleus). In order to avoid confusions, this is now clearly stated (L183-184 in the version with tracked changes).

*Line 129: “obvious”? please explain.*

> We have now replaced the term “obvious” by “noticeable”, and specify the observable changes: “C2-GFP, which is excluded from the nucleolus in the absence of the virus, accumulates in this compartment in infected cells; C3-GFP, on the contrary, is excluded from the nucleolus in the presence of the virus; C4-GFP is depleted from the plasma membrane and accumulates in chloroplasts; and CP-GFP re-localizes from the nucleolus to the nucleoplasm, where it accumulates in unidentified structures.” (L186-191 in the version with tracked changes).

*Line 137 and Fig. 2B: the nucleolar localization is visible not only in C2/CP, but also, although partially, in C2/Rep and C2/C3: this is neither mentioned nor discussed.*

> The exclusion of C2-GFP from the nucleolus is normally visible, although not complete. In our hands, we have not observed a consistent nucleolar accumulation of C2-GFP in combination with any of the viral proteins with the exception of CP, with which the nucleolar localization of the protein becomes extremely apparent. In order to distinguish the effect of CP from the basal status of C2-GFP (or potential minor effects of other viral proteins), we now refer in the text to “a strong accumulation of C2 in the nucleolus” (L197 in the version with tracked changes).

*Line 90 and Fig.1: “Our results show that, strikingly, viral proteins form prevalent pairwise interactions in the context of the viral infection, displaying a high degree of intraviral connectivity, as demonstrated by an array of protein-protein interaction techniques.” This is shown in Fig.1, which merits much deeper explanation and discussion, even in those cases where some data do not go in the direction expected. As an example, compare, in fig. 1, panel D (Summary of viral protein-protein interactions detected by co-immunoprecipitation (co-IP) in the absence or presence of TYLCV), with panel G (Summary of the intra-viral protein-protein interactions). In G which columns of D were taken? Why was it important to put in D both a column “-“ and one “+” TYLCV? Explain.*

> We thank the reviewer for this suggestion. We have now expanded the explanation of the experiments presented in previous Fig. 1 (new Fig. 2), as well as the description and discussion of the results (see L112-174 in the version with tracked changes). We consider an interaction positive in co-IP if this is detected at least in two replicates, with or without the virus; this is now indicated in the figure legend. We have also explained in the text the reasoning behind performing the co-IPs both in the presence and the absence of TYLCV: “The viral infection reshapes the cell environment where the viral proteins coexist: the presence/absence of host proteins, the activation of post-translational modification pathways, or the presence of additional viral proteins might influence the outcome of the tested interactions. Therefore, co-IP assays were performed both in the presence and absence of the virus.” (L130-134 in the version with tracked changes).

*C2/C2 interaction is evident in Y2H (panel C), but is missing in panel G and in panel H: why?*

> The C2 protein has a transactivation domain and therefore behaves as an auto-activator in Y2H assays, as demonstrated by yeast growth when C2 is expressed together with an empty vector control. This is in agreement with previous observations (Hartitz et al., 1999; Lozano-Durán et al., 2011) and is now specifically mentioned in the manuscript (L123-126 of the version with tracked changes).

**Part III – Minor Issues: Editorial and Data Presentation Modifications**

Reviewer #2:

*Fig 2A, virus infection can affect the subcellular localization of C3. It seems that C3 is excluded from nucleolus in the presence of virus infection. Please explain this why.*

> We consistently observe that the viral infection affects the subnuclear localization of C3, causing its nucleolar exclusion. At the moment, we do not know the underlying molecular mechanism nor the biological impact of this change, but these aspects will be the focus of future investigations.

*Title is too big and should more specific.*

> Following the reviewers’ advice, we have now modified the title to make is more specific (“Combinatorial interactions between viral proteins expand the potential functional landscape of the tomato yellow leaf curl virus proteome”).

Reviewer #3:

*Colors in panel G of Fig.2 are not easily distinguishable, probably thicker lines, and/or a modification of colors, can improve visibility.*

> We thank the reviewer for this suggestion. We have now increased the size of this panel in order to improve visibility.

*If there are pieces of this work that are from previous papers of the same authors, this should be clearly stated. One example is the IP-MS work, which apparently is from ref. 36.*

> The original IP-MS experiments were performed as part of a previous work, which is cited in the text (Wang et al., 2017); the original data were re-analyzed in order to search for the viral proteins, and those results are included in this manuscript. This is now clearly indicated (L166-167 in the version with tracked changes). The rest of experimental data have not been previously published.

*Supplementary Table 1 merits a better legend.*

> Following the reviewer’s suggestion, we have modified the figure legend of Supplementary table 1, which now reads “Supplementary table 1. Viral protein-protein interactions detected by AP-MS. The AP-MS experiment is from Wang et al., 2017; data from independent replicates are separated by commas”.
